# Product Review: MicrobeCards

**DOI:** 10.3201/eid0904.030055

**Published:** 2003-04

**Authors:** Kimberly Sessions

**Affiliations:** Emory Center for AIDS Research, Atlanta, GA

As someone whose professional life is split between teaching lay people to understand scientists and teaching scientists how to talk intelligibly to lay people, I am always looking for good teaching aids. Under the right conditions, MicrobeCards are definitely one such aid. MicrobeCards are a deck of 103 palm-sized (6 cm x 9 cm), color-coded flash cards, which collectively provide a surprisingly large amount of accurate, well-organized information about five categories of microbes: gram-positive bacteria, gram-negative bacteria, viruses, fungi, and parasitic organisms.

All cards use the same format. The front is color-coded by microbe category and provides full-color microscopy and clinical images. The back features a schematic of the human body, showing, at a glance, where the microbe causes disease. It also displays a standardized summary of five key features: pathogenesis, immunity, epidemiology, diagnosis, and control. The microbe’s name appears on front and back, and all labeled illustrations on the front are keyed to points in the summary on the back.

The strengths of this product are its portability, comprehensiveness, and decidedly low-tech approach to teaching. Small enough to slip into a shoulder bag, MicrobeCards are crammed with a textbook’s worth of information. Having trouble waking up during the morning commute? Use the next traffic jam to test yourself on *Streptococcus pyogenes*—is the photo on the left acute impetigo or necrotizing fasciitis? Do you want to strike up a conversation with someone in line at the Burger Doodle but can’t think of a way to break the ice? Simply reach in your bag, hand him or her flash card 97, and your new acquaintance test you on the pathogenesis of *Taenia saginata* (answer: “a) Encysted larvae are ingested in undercooked beef. Cysticerci are released, attach to the small intestine by b) a hookless head and grow unto adult worms up to 10 meters long in 3 months. c) Each segment of the worm (proglottid) has male and female sexual organs and is capable of producing over 1,000 eggs. Proglottids are motile and can migrate—for example, from the anus at night.”). The card describes this experience as “disconcerting.”

For science students who would rather hang out in Java Monkey than the library, these low-tech flash cards just can’t be beat (particularly because they are low tech). In fact, marketing the product as MicrobeCards, not MicrobePalmPilot, is a smart move. The detailed images, schematics, and linked text on MicrobeCards would be difficult to encompass on a PDA, and laptops are really just too cumbersome to pull out and use in a traffic jam, while in line, or while sunning at the beach. Furthermore, this reviewer found the tactile learning experience (handling the cards, flipping each one back and forth, and watching the growing pile of “learned” cards) more satisfying than covering the same information by mousing from screen to screen.

Of course, all this convenience comes at a price. Most 20-year-old students may have no trouble reading the text on the back of these cards, but the angels-dancing-on-the-head-of-a-pin sized (33 characters per inch) had this reviewer renaming the pack MicroCards and wishing that ASM Press included a complimentary magnifying glass with every purchase. Furthermore, as flashy as these cards are, a couple of small improvements would have made them more useful than, well, flash cards. Specifically, the microbe category color-coding, which appears only on the front of each card, should appear on the back to minimize the use of color as an accidental clue during memory and recognition drills. For the same reason, the name of each microbe should appear only on the back. I found ignoring the colored rims and covering each name with my thumb a bit awkward as I went through the deck, testing my memory (bad) and the cards’ interest level (excellent).

The ideal users for these cards are undergraduate or graduate students (with really good eyesight) who are taking courses in medical microbiology or infectious diseases. I wouldn’t recommend the cards for high school use as they are written at a graduate reading level. On the other hand, life-long learners like my friend Jill, a self-taught polymath who seems to have equal rapport with both sides of her brain, may also find these cards exactly as advertised: A serious learning tool that’s fun and friendly.

